# Activated Mesenchymal Stem Cells Interact with Antibiotics and Host Innate Immune Responses to Control Chronic Bacterial Infections

**DOI:** 10.1038/s41598-017-08311-4

**Published:** 2017-08-29

**Authors:** Valerie Johnson, Tracy Webb, Annalis Norman, Jonathan Coy, Jade Kurihara, Daniel Regan, Steven Dow

**Affiliations:** Center for Immune and Regenerative Medicine, Department of Clinical Sciences, College of Veterinary Medicine and Biomedical Sciences, Colorado State University, Ft. Collins, CO USA

## Abstract

Chronic bacterial infections associated with biofilm formation are often difficult to resolve without extended courses of antibiotic therapy. Mesenchymal stem cells (MSC) exert antibacterial activity *in vitro* and in acute bacterial infection models, but their activity in chronic infection with biofilm models has not been previously investigated. Therefore, we studied the effects of MSC administration in mouse and dog models of chronic infections associated with biofilms. Mice with chronic *Staphylococcus aureus* implant infections were treated by i.v. administration of activated or non-activated MSC, with or without antibiotic therapy. The most effective treatment protocol was identified as activated MSC co-administered with antibiotic therapy. Activated MSC were found to accumulate in the wound margins several days after i.v. administration. Macrophages in infected tissues assumed an M2 phenotype, compared to untreated infections which contained predominately M1 macrophages. Bacterial killing by MSC was found to be mediated in part by secretion of cathelicidin and was significantly increased by antibiotics. Studies in pet dogs with spontaneous chronic multi drug-resistant wound infections demonstrated clearance of bacteria and wound healing following repeated i.v. administration of activated allogeneic canine MSC. Thus, systemic therapy with activated MSC may be an effective new, non-antimicrobial approach to treatment of chronic, drug-resistant infections.

## Introduction

Chronic implant and wound infections continue to be a major source of morbidity and mortality in patients, driven in part by the increasing prevalence of drug-resistant bacteria and by the greater incidence of diseases such as diabetes mellitus that predispose to chronic infections^1–4^. Infections characterized by the development of bacterial biofilms, which often develop on the surface of implants such as catheters, orthopedic devices, or partially devitalized tissues, are particularly difficult to manage with antibiotic therapy alone, often requiring weeks to months of continuous therapy. Despite aggressive antibiotic therapy, in many cases biofilm-infected devices or implants must be removed to fully resolve these chronic infections. Thus, there is a strong unmet need for alternatives to conventional antibiotic therapy for management of chronic infections^5^. Current alternative therapies for management of chronically-infected wounds include the use of antibiotic impregnated implant materials or biological scaffolds, administration of biofilm disrupting agents, and administration of immunotherapy together with antibiotics^6, 7^.

It has been previously established that mesenchymal stem cells (MSC) exhibit antimicrobial properties. These effects have been demonstrated to be both direct and indirect^8^. For example, MSC have been shown to secrete antimicrobial peptides, including cathelicidins, lipocalin-2 and beta-defensins^9–14^. In some studies, secretion of the cathelicidin LL-37 by MSC was shown to be enhanced by bacterial products, indicating that MSC can upregulate antimicrobial activity in the presence of infection^9^. Mesenchymal stem cells have also been shown to interact with the host innate immune system to increase antibacterial activity. For example, several studies have demonstrated increased phagocytosis and killing properties of monocytes and neutrophils following exposure to MSC-secreted factors, and it has been observed that MSC also suppress inflammation in sepsis models^15–26^. The potential of MSC therapy for systemic infection management is also supported by a recent study which found that MSC acted synergistically with antibiotics to enhance survival in a mouse model of sepsis^24^. Other studies have demonstrated wound healing properties of MSC, including stimulation of angiogenesis, activation of resident stem cell populations, reduction in inflammation, recruitment of immune cells, and suppression of scarring^27–31^.

Other studies have examined whether pre-activation of MSC with Toll-like receptor (TLR) agonists or cytokines can alter their antimicrobial properties^32^. For example, it was found that activation of MSC with TLR3 ligands triggered release of factors that enhanced neutrophil survival^17, 33^. In addition, TLR3 agonists were shown to stimulate greater release of neutrophil chemokines than other TLR agonists and have also been shown to stimulate the migratory properties of MSC^33–36^. Although other TLR agonists have been examined for purposes of MSC activation, previous studies in out lab demonstrate increased bacterial killing only with MSC preactivation with TLR3 and TLR4 agonists (data not shown). These observations suggest that it may be possible to manipulate the antimicrobial and immunological properties of MSC *ex vivo* prior to therapy to stimulate greater clinical benefit in the setting of chronic infections. Additionally, these findings would suggest that preactivation of MSC with TLR3 agonists would provide the ideal combination of antimicrobial and immune stimulating effects.

The utility of treatment with MSC has been demonstrated in animal models of acute infection and wound healing^10, 12, 25, 26, 28–31, 37–39^. However, to date there is no published experimental or clinical evidence that administration of activated MSC can be effective for infection management in two common clinical scenarios: treatment of chronically-infected implants, and treatment of infections involving multidrug resistant bacterial infections^40^. Therefore, we used a murine *S aureus* implant infection model to assess the effect of systemic treatment with TLR-activated MSC and concurrent antibiotic therapy for treatment of chronic implant infections. These studies were conducted to optimize treatment conditions and to elucidate microbiological and immunological mechanisms of activity. In addition, proof-of-concept studies of combined activated MSC and antibiotic therapy were conducted to assess the efficacy of activated MSC and antibiotic therapy in pet dogs with spontaneous multi-drug resistant infections.

The findings reported here indicated that systemic administration of activated MSC was effective in controlling chronic implant infection when co-administered with antibiotic therapy, and that the effect involved both direct and indirect antibacterial and immunological mechanisms. Importantly, the pilot studies in dogs with spontaneous chronic infections demonstrated that repeated i.v. administration of activated allogeneic MSC could be employed effectively with antibiotics for treatment of chronic, drug-resistant infections. These findings suggest that systemic treatment with activated MSC may represent an important new approach to managing chronic infections with multi-drug resistant bacteria.

## Materials and Methods

### Bacteria and biofilm preparation

The Xen36 strain of *Staphylococcus aureus*, expressing the luciferase gene for bioluminescent imaging, was purchased from Caliper Life Sciences (Perkin Elmer, Santa Clara, CA). Bacteria were propagated in LB medium (BD Falcon) and utilized to coat mesh implanted material during the log phase of growth.

### Mice

Mice used in these studies included 8–12 week old CD-1 mice, male and females, and were purchased from Charles River Laboratories. Adipose tissues from transgenic mice expressing GFP under the ubiquitin promoter were kindly provided by Dr. William Janssen, National Jewish Hospital, Denver, CO^41^. CCR2-gfp reporter mice (on the C57Bl/6 background) were provided by Dr. Eric Pamer (Memorial Sloan Kettering, NY)^42^. Animals were maintained in sterile microisolater cages under controlled temperature and humidity and were fed sterilized food and water. All procedures involving mice in this study were approved by the Institutional Animal Care and Use Committee at Colorado State University.

### Clinical trial design for wound infection study in pet dogs

Dogs enrolled in a pilot clinical study of MSC therapy for wound infection management were client-owned animals that presented to the Colorado State University Veterinary Teaching Hospital for treatment of multidrug resistant chronic wound infections. These studies were approved by the Colorado State University Institutional Animal Care and Use Committee and by the Clinical Review Board at the Veterinary Teaching Hospital. Inclusion criteria involved presence of a documented infection with a multi-drug resistant organism in a site where cultures could be obtained on a regular basis for quantitative analysis. In addition, the infection must have failed conventional treatment for a period of greater than 2 weeks. To fulfill this criteria, animals must have failed treatment with antibiotics and surgical debridement as evidenced by continued symptoms of infection and positive culture of organisms at the site. Animals were excluded from the trial if there was any evidence of systemic disease in which treatment with MSC may be contraindicated, such as neoplasia, or that may affect immune function, such as Cushing’s disease or organ failure. Animals were also excluded if the site of the infections was such that obtaining quantitative cultures would not be possible.

Dogs enrolled in the trial were treated with allogeneic MSC pre-activated with 10 ug/ml of poly I:C (InVivoGen, San Diego CA) × 1 hr at a dose of 2 × 10^6^ cells per kg body weight. Cells were then washed 3× in DPBS (Sigma-Aldrich, St. Louis, MO) and re-suspended in DPBS at a volume of 10 ml (30 × 10^6^ cells or less), 15 ml (30–45 × 10^6^ cells) or 20 ml (>45 × 10^6^ cells). Sodium heparin (Fresenius Kabl USA, Lake Zurich IL) was added immediately prior to injection at 200 IU per 10 ml of cell solution. Cells were delivered by intravenous (i.v.) infusion over 15 minutes. Heart rate and respiratory rate were monitored during and for 10 minutes after infusion. Antibiotic therapy was kept consistent for the duration of the study (and consisted of the last antibiotic the animals had been treated with before concluding failure of antibiotic therapy) and continued for 1 week after the last injection was administered. Dogs were treated with MSC every 2 weeks for a total of 3 treatments, and quantitative cultures were obtained prior to each treatment. Cultures were obtained by fine needle aspirate of 4 quadrants of the infected area. The aspirates were placed in 1 ml of TSB (trypticase soy broth, Sigma Aldrich) and quantitative cultures obtained by 10 fold dilution in PBS and plating on LB agar (Luria Broth, Sigma Aldrich). Dogs were considered for additional MSC injections if there was a significant decrease in bacterial infection (but not full bacterial clearance) as evidenced by a significant decrease in number of bacteria in the wound or eradication of 1 or more but not all species of bacteria present at the infection site. Two weeks after the final treatment, quantitative cultures were again performed, and patients were then monitored by follow up phone calls every 2–3 months. Dogs were considered to have a positive response if clinical signs improved and bacterial counts decreased significantly over the treatment interval. The infection was considered eradicated if the animal had more than 2 negative cultures at least 2 weeks apart and the infection site was fully healed with resolution of clinical signs.

### Generation of adipose-tissue derived MSC

Adipose tissue-derived MSC were generated from abdominal and inguinal adipose tissues collected from mice. To prepare MSC cultures, adipose tissues were minced under sterile conditions, incubated in a 1 mg/ml solution of collagenase (Sigma-Aldrich, St. Louis, MO) at 37 °C and 5% CO_2_ for 30 minutes, and then triturated. The cell suspension was then centrifuged at 1,050 X G to pellet the stromal vascular fraction (SVF), which was resuspended in MSC culture medium. MSC culture medium consisted of low glucose DMEM (InVitrogen/Gibco, Carlsbad, CA) supplemented with essential and non-essential amino acids (InVitrogen/Gibco), glutamine (InVitrogen, Gibco), 15% heat-inactivated FBS (Cell Generation, Ft. Collins, CO), and penicillin and streptomycin solution (InVitrogen/Gibco). Cells in medium were allowed to adhere in tissue culture flasks (BD Falcon, Bedford, MA) for 72 hr, after which the non-adherent cells were removed and fresh medium was added. When cells reached 80–90% confluence, they were passaged using trypsin-EDTA solution (InVitrogen/Gibco). Cells for i*n vivo* experiments were used at passage 2–3 for all experiments. The cell surface phenotype of MSC was determined by flow cytometry, and the ability of the MSC to undergo tri-lineage differentiation was assessed as described previously^43^.

Canine MSCs were generated from adipose tissue obtained from the inguinal region of healthy, young purpose bred research hounds. The adipose tissue was frozen in 1 gm aliquots in freezing media consisting of 75% FBS, 15% MSC medium, and 10% DMSO at −80 degrees within 2 hours of sample collection. Freshly-cultured MSC were generated from frozen adipose tissue aliquots for each treatment, and the cells used between passages 2 and 5 for all dogs. The cryopreserved adipose tissue was thawed and rinsed in DPBS twice. Subsequently the tissue was minced, digested in collagenase, the SVF collected and culture-expanded as described above for mice. At the time of adipose tissue collection, blood was obtained from each donor dog for a complete blood count and chemistry profile. Additional testing for infectious disease included serology to test for *Anaplasma phagocytophilum*, *Borrelia burgdorferi*, *Dirofilaria immitis*, *Ehrlichia canis* (4DX, Idexx laboratories), and PCR was performed to detect the presence of *Hemoplasma spp*, *Ehrlichia spp*, *Bartonella spp*, and *Rickettsial spp* at the state veterinary diagnostic laboratory. All donors had normal CBC and biochemistry profiles and were negative on all infectious disease testing. In addition, each stem cell culture was tested for bacterial, fungal, and mycoplasma contamination at the time of stem cell administration using in house cultures (blood agar, McConkey agar, Sabourad Dextrose agar – BD Falcon, and mycoplasma agar – Udder Health systems, Bellingham WA). Bacterial cultures were incubated at 37 °C for 48 hr, mycoplasma cultures were incubated at 37 °C, 5% CO_2_, for 7 days, and fungal cultures were incubated at room temperature in the dark for 30 days. No contamination was detected in any culture during the study. Cell surface phenotype was assessed via flow cytometry and tri-lineage differentiation was performed as previously described^43^.

### Staphylococcus infected mesh model and IVIS imaging

To assess the effects of MSC on chronic wound infections, we utilized a chronic *S. aureus* implant infection model previously developed by our laboratory^44^. Briefly, surgical mesh material (Covidien, Mansfield MA) was cut into 0.5 mm squares, then incubated with Xen36 bacteria in sterile 10 mm petri dishes for 24 h with gentle rotary shaking at 37 °C. The mesh was then washed in PBS prior to implantation into subcutaneous tissues. Mice were anesthetized with isoflorane, mesh was inserted surgically into a skin flap created in the subcutaneous space over the shoulder, and then the wound was closed with surgical staples. Mice were imaged using an IVIS Spectrum live animal imager (Caliper Life Sciences) to detect and quantify bacteria growing on implanted mesh. We previously demonstrated that the luminescent signals obtained from mice with implants of Xen36 *S. aureus* were linearly correlated with the bacterial burden, as determined by conventional bacterial CFU determination^44^.

IVIS imaging of injected MSC. To track the distribution of MSC *in vivo*, cells were labeled with the far red dye DiR (DiIC18(7) (1,1′-Dioctadecyl-3,3,3′,3′-tetramethylindotricarbocyanine iodide) (Molecular Probes and ThermoFisher Scientific, Waltham, MA) by incubation at a concentration of 2.5 uM in PBS at 37 °C for 30 min, followed by washing and resuspension in DPBS prior to injection. Animals were injected with 1 × 10^6^ labeled MSC in 200 μl DPBS with heparin (100 IU/ml) by the tail vein route, and mice were then briefly anesthetized and subjected to IVIS imaging at the indicated time points. Images were acquired by the IVIS imager, and images were reconstructed using IVIS image software, which was also used to quantitate the density of cells in vivo, using reference images produced with known quantities of DiR-labeled MSC.

### Bacterial quantitative culture from *in vitro* assays and wound samples

Bacteria numbers were quantitated by plating serial, log10 dilutions of culture supernatants obtained by gentle pipetting, and then plating on LB agar quadrant plates (Sigma-Aldrich) and counting bacterial colonies 24 hr after culture at 37 °C. The log_10_ CFU was determined numerically from plate counts. For quantitation of bacteria in wound tissues of mice, tissues at the infection site were collected and weighed and then homogenized in PBS using a tissue homogenizer. The supernatants of these homogenates were then serially diluted and plated, and colonies with the typical appearance of *S. aureus* were quantitated.

For determination of the bacterial burden in wound tissues of dogs in the clinical trial, 4-quadrant fine needle aspirates were collected from around the wound perimeter, inoculated into LB medium, then serially diluted and plated on LB plates for quantitation and Trypticase Soy Blood Agar and MacConkey plates (BD Falcon, San Diego CA) for bacterial growth and enumeration. Plates were incubated for a minimum of 48 hr at 37 °C to confirm absence of bacterial growth. Animals with septic arthritis underwent aspiration of the affected joint under sedation every 2 weeks during the study period. Joint fluid was also evaluated cytologically. Bacteria were speciated by morphology and biochemical testing, and bacterial CFU were determined by manual counting. Bacterial sensitivities were performed by the Colorado State veterinary diagnostic laboratory (200 West Drake Road, Fort Collins CO 80523).

### Bacterial killing assay

To assess the ability of MSC to kill bacteria, cells or conditioned medium (CM) from MSC were utilized. CM was generated by plating 5 × 10^5^ cells per well in a 24 well plate with 500 ul per well of antibiotic free media and incubating at 37 °C. Conditioned media was collected 24 h after the cells were plated and were immediately frozen at −80 °C and thawed immediately before use. MSC or CM were inoculated with log phase *S. aureus* cultures, typically at a concentration of 1 × 10^5^ CFU bacteria per ml of CM or per ml of cells in 24-well plates, in complete MSC culture medium, but without antibiotics. Cells in culture were inoculated with bacteria at a multiplicity of infection (MOI) of 10:1 (bacteria per cell). Co-cultures were incubated at 37 °C for 3 hr, then numbers of viable bacteria was determined.

To assess the potential role of cathelicidins in MSC-mediated bacterial killing activity, MSC CM was incubated with an anti-cathelicidin antibody (Abcam, San Francisco CA) at 50 μg/ml for 10 minutes prior to addition of bacteria. An irrelevant rabbit IgG (Abcam, San Francisco CA) was used at the same concentration in parallel cultures as a control for specific cathelicidin activity neutralization.

The ability of MSC or MSC CM to augment antibiotic activity was performed by assessing bacterial killing activity or MSC or CM, with or without the addition of low concentrations of cefazolin. In addition, studies of enhancement of antibiotic activity were also done using recombinant LL-37 (InVivoGen), with or without low concentrations of cefazolin.

### Neutrophil bacterial phagocytosis assay

Neutrophils were collected from the peritoneal cavity of mice 24 hr following intra-peritoneal injection of 1 ml aged thioglycollate, as described previously^45^. For assessment of the effects of MSC CM on neutrophil phagocytosis, 1 × 10^6^ neutrophils were incubated with MSC-CM for 1 hr at 37 °C. The neutrophils were washed twice in PBS, seeded in 4-well chamber slides (BD Falcon), inoculated with log phase *S. aureus* at an MOI of 1:1 for 30 min at 37 °C, and then treated with gentamicin (300 ug/ml) for 30 minutes to eliminate extracellular bacteria. The neutrophils were then washed twice with PBS, fixed with 1% paraformaldehyde, and immunostained for detection of intracellular *S. aureus* using an anti-staphylococcus antibody (Genway Biotech, San Diego CA) and with a fluorescently conjugated antibody to Ly6G (eBioscience, San Diego CA). The relative numbers of phagocytosed *S. aureus* per neutrophil were determined by microscopy with an Olympus IX-83 confocal microscope, using appropriate lasers and filters and quantitated by image analysis software (Image J).

### Monocyte migration assay

Monocytes were collected from the peritoneal cavity of mice 72 h after intra-peritoneal administration of 1 ml aged thioglycollate, as described previously^45^. Monocytes collected from the peritoneal cavity by lavage with PBS were washed and then resuspended in complete medium. Migration was measured using Boyden chambers (BD Falcon) with 8 um pore diameter. Monocytes (2.5 × 10^5^ cells per well) were added to the top chamber in complete medium, MSC CM was added to the bottom chamber, and the assay was run for 4 hr at 37 °C. Recombinant mouse CCL2 (100 ng/ml; R&D Systems Inc., Minneapolis, MN) was used as a positive chemokine control. The numbers of migrated cells were determined by first removing cells from the top of the membrane with cotton swabs, then removing the filter membrane, staining with crystal violet, and manually counting the number of monocytes adherent to the bottom of the membrane, averaging at least 5 random high power fields per sample. Data were displayed as average number of migrated cells per membrane per treatment condition.

### Tissue histology

Wound tissues were collected, immersion fixed in 10% neutral buffered formalin, and paraffin-embedded for routine histological processing. Tissues were sectioned at 5 μm and stained with hematoxylin and eosin (H&E) for histopathological evaluation. Images were captured using an Olympus IX-83 confocal microscope and Olympus SC30 camera.

### Immunohistochemistry and fluorescent imaging

For immunofluorescence evaluation of MSC trafficking to sites of infection, wound tissues were immersion fixed in 1% paraformaldehyde-lysine-periodate fixative (1% paraformaldehyde in 0.2 M lysine-HCL, 0.1 M anhydrous dibasic sodium phosphate, with 0.21% sodium periodate) for 24 hr at 4 °C. Following fixation, tissues were placed in a 30% w\v sucrose solution for 24 hr at 4 °C, prior to embedding and freezing in O.C.T. compound (Tissue Tek, Sakura Finetek USA, Inc., Torrance, CA). Embedded tissues were sectioned to a thickness of 5 μm for immunostaining. Tissue sections were immunostained with a rabbit antibody to GFP (Life Technologies) to enhance GFP signal intensity, followed by incubation with a donkey anti-rabbit IgG antibody, conjugated to AF647 (Jackson ImmunoResearch, West Grove, PA). To identify macrophages with an M1 functional phenotype, tissues were permeabilized with 0.01% Triton X and immunostained using an anti-iNOS antibody (Thermo Fisher Scientific, Waltham, MA), followed by incubation with a secondary donkey anti-rabbit cy3 conjugated antibody (Jackson ImmunoResearch). Macrophages with an M2 phenotype were identified using anti-arginase antibody (Santa Cruz Biotechnology Inc., Dallas, TX) followed by incubation with a secondary donkey anti-goat antibody conjugated to AF647 (Jackson ImmunoResearch).

Immunofluorescent staining for cathelicidin production by MSC was performed by seeding 2 × 10^5^ MSC onto each well of a 4-chamber slide and incubating overnight at 37 °C followed by permeabilization with 0.01% TritonX with subsequent incubation with a rabbit anti-cathelicidin antibody (Abcam, San Francisco CA) followed by incubation with a donkey anti-rabbit IgG antibody conjugated to AF555 (Jackson ImmunoResearch).

For imaging of MSC labeled with DiR or DiD dyes, the tissues were processed similarly and examined with an Olympus IX-83 confocal microscope, using appropriate lasers and filters. Image analysis was performed using CellSens software (Olympus).

### Cytokine assays

Cytokine concentrations in supernatants were assayed using commercial ELISA assays performed according to manufacturer’s directions. The ELISA kit for murine CCL2 detection was purchased from R&D Systems Inc.

### Statistical analyses

Statistical comparisons between data sets with two treatment groups were done using nonparametric t-tests (Mann-Whitney test). Comparisons between 3 or more groups were done using ANOVA, followed by Tukey multiple means post-test. Tests for synergy in bacterial killing were performed using a 2-way ANOVA, according to a previous approach^46^. Statistical analyses were performed using Prism5 software (GraphPad, La Jolla, CA). For all analyses, statistical significance was determined for p < 0.05.

### Study approval

All studies involving animal use were approved by the Colorado State University Institutional Animal Care and Use Committee and all experiments were performed in accordance with relevant guidelines and regulations. Use of pet animals enrolled in clinical trials was additionally reviewed by the Clinical Review Board at the Veterinary Teaching Hospital at Colorado State University. Prior to enrollment of an animal in the clinical trial, owner informed consent was obtained.

The datasets generated during the current study are available from the corresponding author on reasonable requests.

## Results

### Effects of i.v. treatment with activated MSC on wound bacterial burdens

Previous studies have demonstrated that topical administration of MSC can improve wound healing and infection control^10, 29, 31^. Other studies have evaluated the effectiveness of MSC administration for control of acute infections, including pneumonia and sepsis, in mouse infection models^9, 19, 13, 24, 26, 47–50^. However, the use of MSC to treat chronic bacterial infections associated with biofilms has not been evaluated previously. We used a biofilm model that utilizes the s.c. implantation of surgical mesh coated with luciferase-expressing *S. aureus* to assess the effects of MSC treatment on chronic bacterial infections^44^. In this particular model, treatment with antibiotics is ineffective in reducing bacterial infection at the wound site. Six different treatment groups of mice (n = 5 per group) were used in the study: 1) untreated control animals, 2) animals treated with antibiotics only (orally-administered amoxicillin-clavulanic acid), 3) animals treated with MSC only, 4) animals treated with activated MSC only, 5) animals treated with MSC plus antibiotics, and 6) animals treated with activated MSC plus antibiotics. Mesenchymal stem cells were administered i.v. by tail vein injection 3 times, at 3-day intervals, at a dose of 1 × 10^6^ cells per injection per mouse. The systemic route of administration of MSC was selected because initial studies determined that local s.c. injection of activated MSC around the site of the infected mesh, in combination with antibiotic therapy, was ineffective in clearing infections (data not shown).

**Figure 1:**
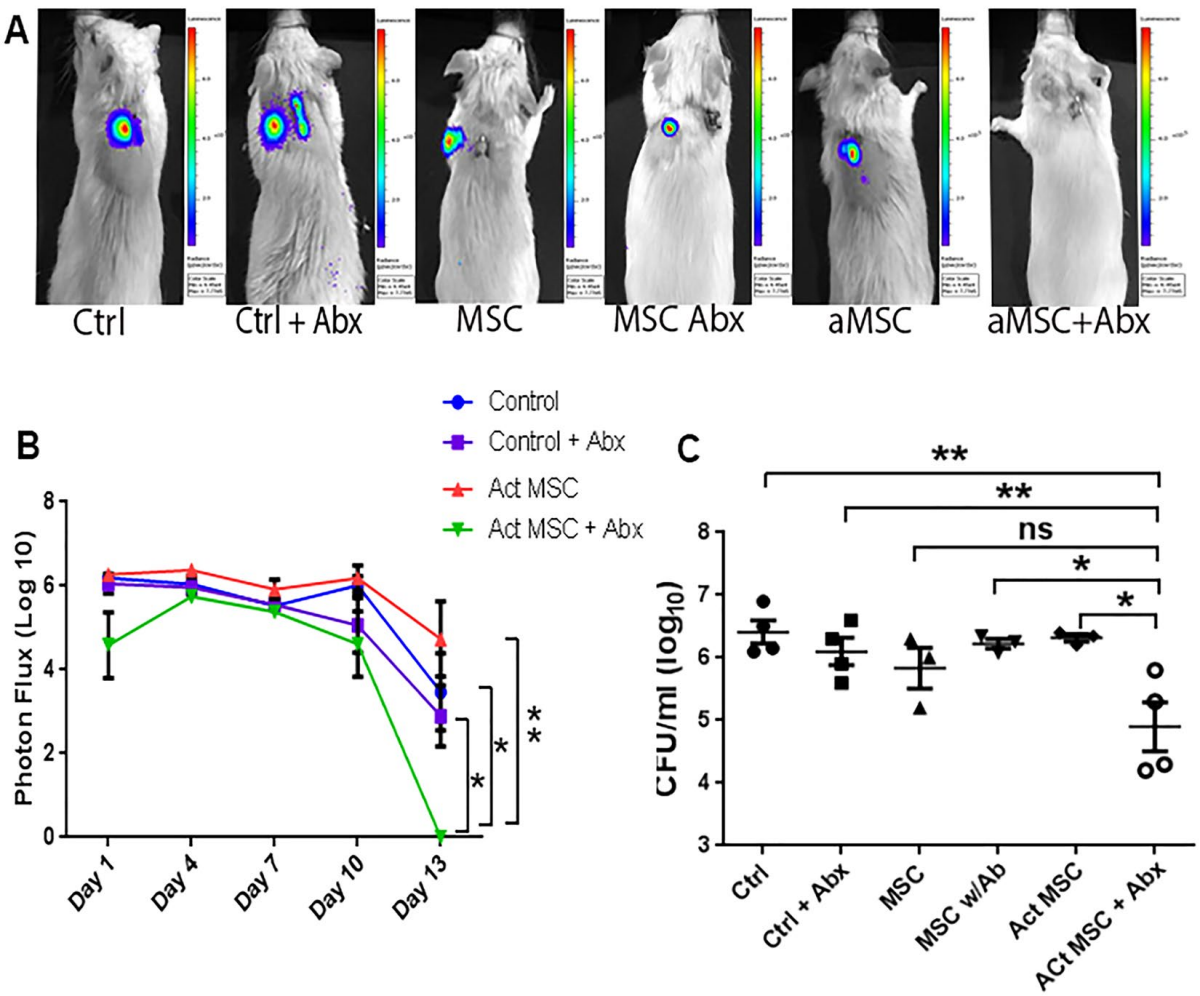
Effects of MSC and antibiotic administration on bacterial infection in mouse chronic wound implant infection model. CD-1 mice (n = 5 per group) were implanted with surgical mesh on which biofilms has been established with a luciferase-expressing strain (Xen36) of *S. aureus*, as described in Methods. On day 2 after implant placement, mice were randomly assigned to the following treatment groups: untreated controls; treatment with antibiotic (amoxicillin-clavulanic acid, continuous treatment in drinking water) only; treatment by i.v. administration of untreated MSC; treatment by i.v. administration of activated MSC; treatment by administration of untreated MSC plus antibiotics; and treatment with activated MSC plus antibiotics. Mice were imaged by IVIS bioluminescence imaging every 2–3 days following mesh implantation to assess the effects of treatment on bacterial burden. In (**A**), representative IVIS images of wounds in one mouse of each treatment group (n = 5 animals per group) are depicted. Quantitative mean photon intensity from each group of treated animals over time is depicted in (**B**). Similar results were obtained in 2 additional experiments. In (**C**), the mean bacterial burden in wounds were determined on day 14 of infection by quantitative counting, as described in Methods, and compared statistically by ANOVA and Tukey multiple means comparison. *Denotes p < 0.05.

The results of *in vivo* bioluminescence imaging and direct bacterial counts revealed that administration of antibiotics or MSC alone (activated or resting) did not significantly reduce the bacterial burden at the wound site (Fig. 1). Likewise, the combination of antibiotic therapy and administration of non-activated MSC was also ineffective (Fig. 1). The combination of TLR3 ligand-activated MSC with antibiotic therapy was found to be the only treatment that significantly reduced the bacterial burden at the wound site, based on both bioluminescence imaging and direct bacterial enumeration by convention culture (Fig. 1A–C). Previous studies have also reported that activation of MSC with the TLR3 ligand polyinosinic polycytidylic acid (polyI:C) increased MSC antibacterial activity in mouse sepsis models^51^. Thus we conclude that systemic administration of MSC activated *in vitro* prior to administration interacted synergistically with antibiotic therapy to efficiently reduce bacterial numbers in chronic wounds associated with biofilms.

### Impact of MSC treatment on wound histology

**Figure 2:**
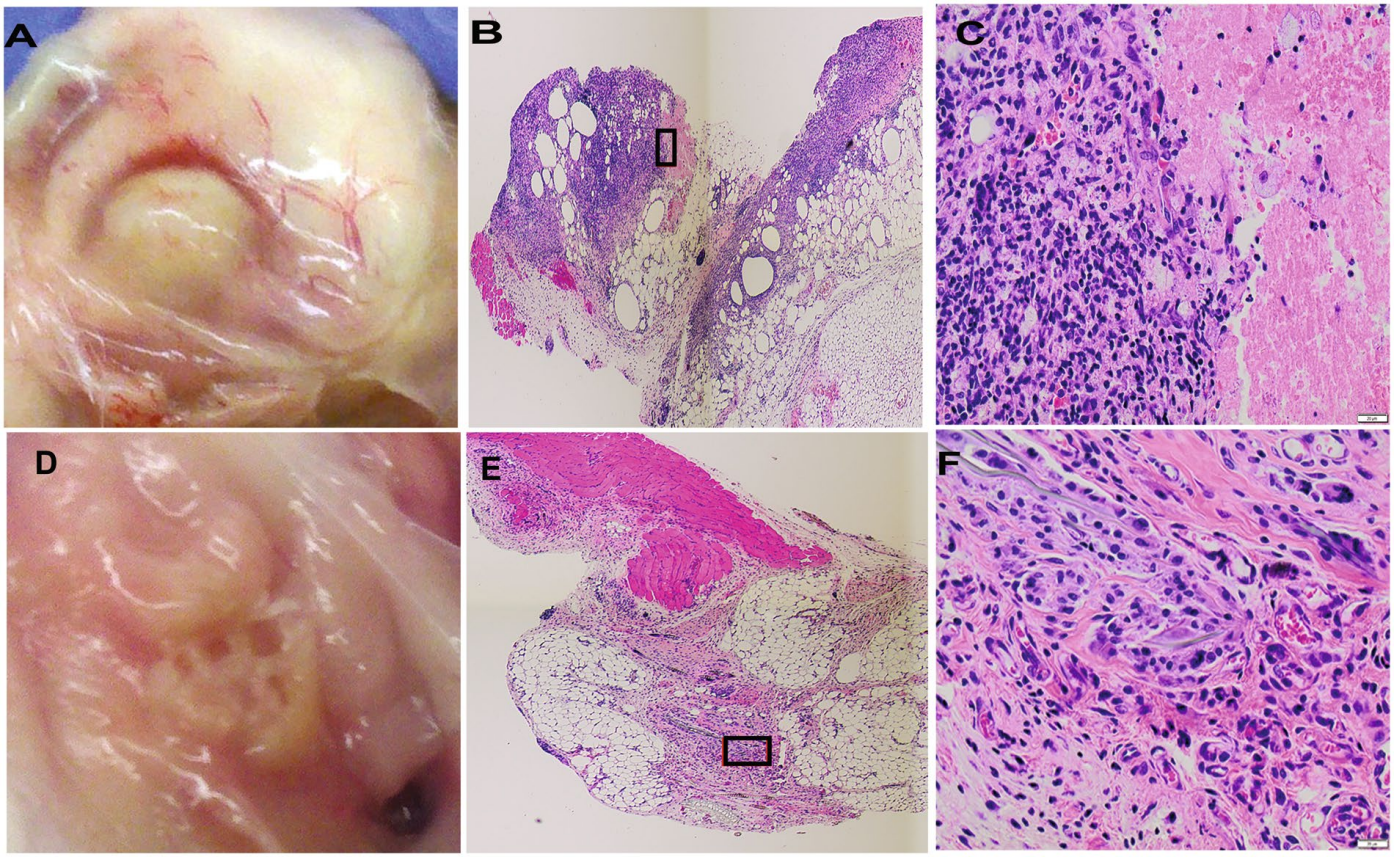
Effects of activated MSC administration on wound healing in mouse chronic infection model. Mice with *S. aureus* infected mesh implants were treated as described in Fig. 1. At the completion of the study (day 14), mice were euthanized and skin was dissected to reveal the implant site. In (**A**), representative photograph of a wound treated with antibiotics only, and corresponding representative photomicrographs of H&E sections (**B**,**C**) revealing suppurative inflammation at the wound site. In (**D**) representative photograph of a wound in a mouse treated with activated MSC plus antibiotics, with implanted mesh visible in wound bed. In (**E**,**F**) representative photomicrographs of (H&E) sections from a mouse treated with activated MSC plus antibiotics, revealing mild monocytic inflammation.

Histologically, infected tissues from the implant infections in antibiotic-only treated animals were characterized as chronic, purulent inflammation with abscess formation with intense neutrophilic infiltrates **(**Fig. 2A–C). In contrast, infected tissues from animals treated with activated MSC and antibiotics contained primarily a mild monocytic cellular infiltrate (Fig. 2D–F). Grossly, infected tissues from animals treated only with antibiotics contained large abscesses centered around the implanted mesh material (Fig. 2A). Conversely, infected tissues from animals treated with activated MSC plus antibiotics were relatively free of purulent material and reactive tissues, such that implanted mesh could be readily visualized in the implanted tissues (Fig. 2D). These findings suggested that systemic administration of MSC was also associated with substantial improvement in infected tissue healing.

### Migration of MSC to sites of infection following i.v. administration

**Figure 3:**
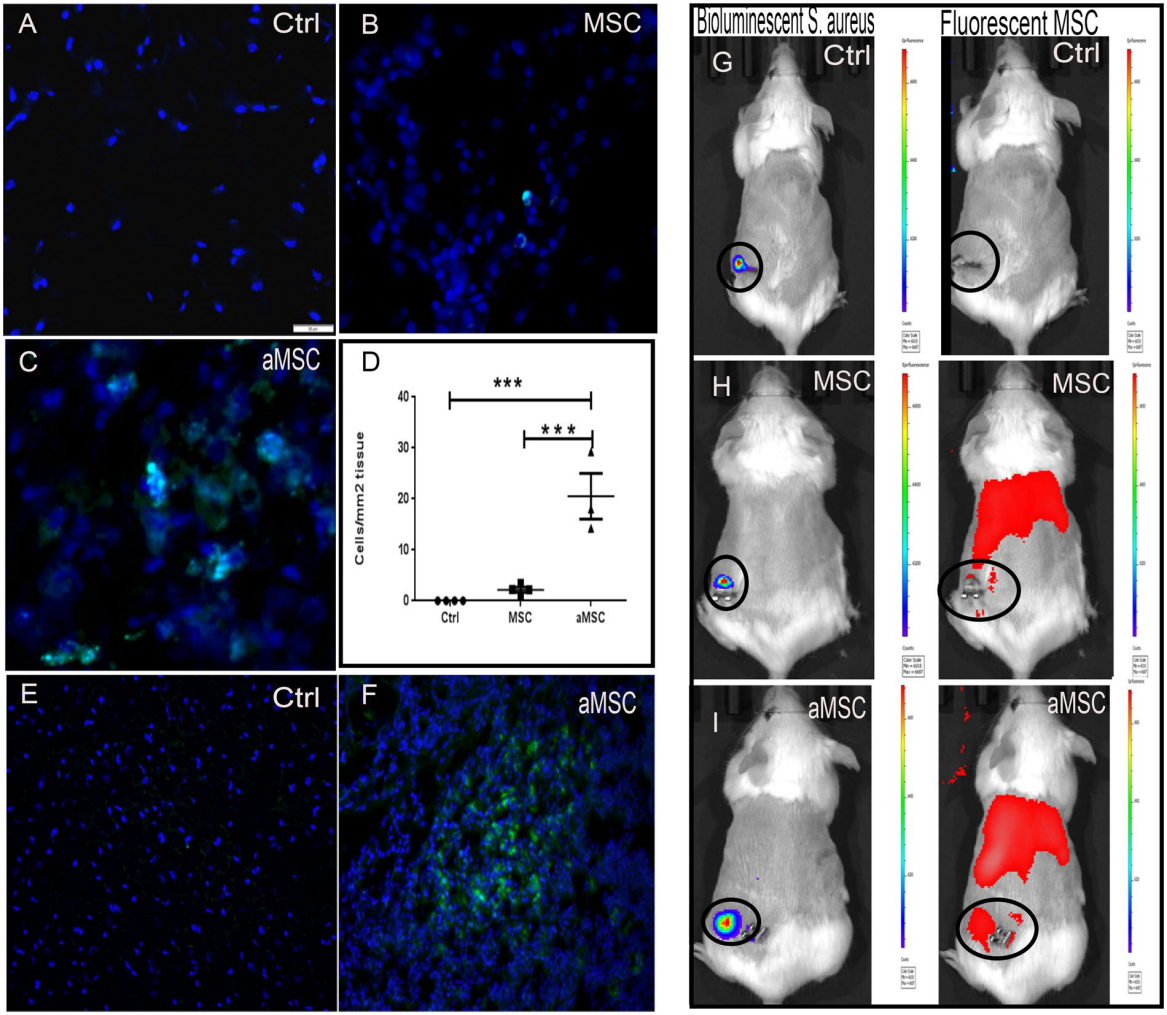
Migration of labeled MSC to infected wound sites following i.v. administration. In (**A**–**C**) representative wound tissues from animals receiving no MSC (**A**), resting MSC (**B**) and activated MSC (**C**). MSC were labeled with the fluorescent membrane dye DiD and detected via fluorescent microscopy (DiD^+^ cells light blue in these images). Animals were injected with labeled MSC 24 hours after implantation of infected mesh and received a second injection 3 days later. Animals were sacrificed 2 days after the second injection and tissues were evaluated histologically. Quantitative counts of DiD^+^ cells revealed significantly more cells present in tissues from animals receiving activated MSC (**D**). All animals in this study received antibiotics. Counts were compared using ANOVA with *depicting p < 0.05. In (**E**,**F**), wound tissues from a control animal and an animal injected i.v. with activated GFP-transgenic MSC to allow detection in wound tissues. Cells were administered 2 separate injections 3 days apart, and 48 h later, the wound tissues were collected and evaluated by fluorescence microscopy for detection of GFP + cells (**F**). In other studies, MSC (activated or non-activated) were labeled with the fluorescent dye DiR, and live mice were imaged using an IVIS imager (**G**–**I**). In the first 24 h after injection, DiR + cells were detected in the lung and spleen. By 48 h post-injection, DiR + cells were apparent in the region of the infected wound. The cells accumulated at the wound sites and images were taken 3 days after the third MSC injection (**H**,**I**). There were also more activated DiR + cells localized in the region of the wound than in mice injected with non-activated cells. Similar results were obtained in one additional animal study.

Previous studies have found that the majority of MSC injected i.v. initially lodge in the lungs, therefore, we used several studies to investigate whether MSC would reach in fact be capable of reaching the site of chronic infection in our model^52^. In one study, mice were injected with MSC generated from the adipose tissues of GFP transgenic donor mice to allow for *in vivo* tracking^41^. For this study, animals with established implant infections were injected twice (3 days apart) i.v. with activated gfp-MSC (1 × 10^6^ cells per injection), starting on day 2 after the infected mesh was implanted. After 2 i.v. injections of gfp-MSC, infection tissues were excised 48 hr after the last injection and immunostained for detection of gfp^+^ cells. Substantial numbers of injected gfp^+^ MSC were observed in the infected tissue margins of treated mice (Fig. 3E,F), thereby providing evidence that i.v. injected MSC were in fact able to migrate efficiently to the infection site. Mice were also injected with DiD-labeled MSC (both activated and non-activated) for live animal imaging assessment of the effect of activation on MSC migration. We found that activated MSC accumulated to a significantly greater degree in infected tissues than non-activated MSC, as assessed by confocal microscopy **(**Fig. 3A–D).

MSC were labeled *in vitro* with the far-red fluorescent membrane dye DiR (Molecular Probes) for live animal imaging (Thermo Fisher Scientific). Mice with s.c. implanted *S. aureus* infected implants were injected i.v. at 3-day intervals with DiR-labeled MSC (1 × 10^6^ cells per injection) for a total of 4 injections and imaged daily (Fig. 3G–I). We found that DiR-labeled MSC could be readily detected in the lungs within hours of injection followed by the appearance of labeled MSC in the spleens of mice within 24 hr of injection. Beginning at 48 hr after injection, DiR-labeled MSC could also be detected accumulating around the margins of infection sites in treated mice (Fig. 3G–I). The number of MSC that accumulated around infected implants increased over time during the 20 days of monitoring. We concluded therefore that i.v. injected MSC did in fact traffic efficiently to infected implants, likely recruited in response to chemokines produced by inflamed tissues, and that activated MSC migrated more efficiently than non-activated cells.

The chemokine stromal cell derived factor -1 (SDF-1, or CXCL12) is a major chemokine regulating MSC recruitment, and high levels of SDF-1 are produced by inflamed tissues^53–56^. To assess the effects of MSC activation on their migratory behavior, we used Boyden chambers to assess MSC migration to an SDF-1 gradient. These studies revealed that poly I:C activation significantly increased MSC migration to the SDF-1 stimulus and that activation was associated with significant upregulation of CXCR4 expression (data not shown). Thus, enhanced migration may explain in part why activated MSC are more effective than resting MSC for treatment of chronic infections.

### Antimicrobial activity of MSC and interaction with antibiotics

**Figure 4:**
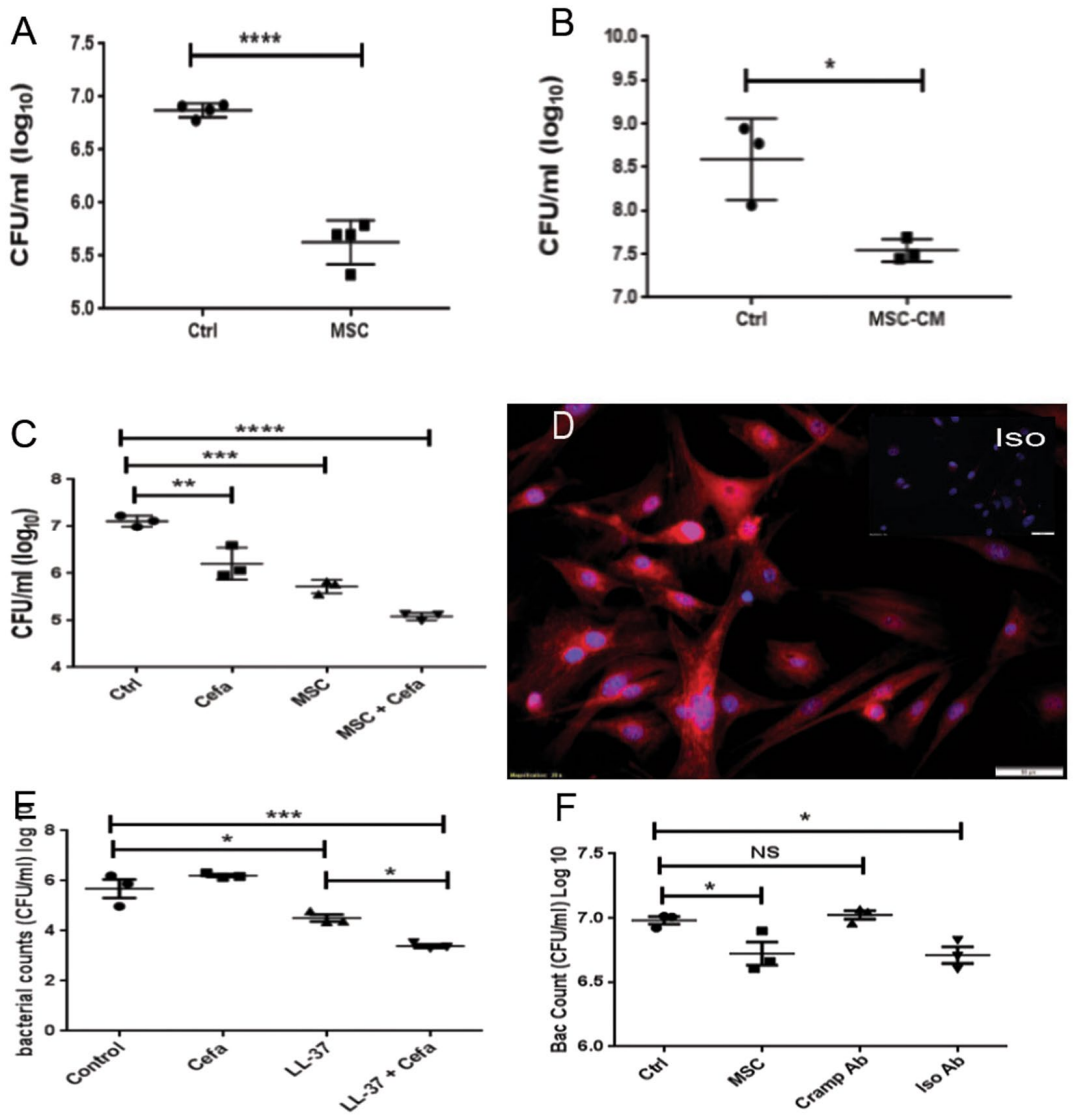
Bacterial killing by MSC mediated in part by the antimicrobial peptide Cramp. The ability of MSC to kill *S. aureus* was assessed by co-culture of bacteria directly with MSC (**A**) or with MSC CM (**B**). MSC (5 × 10^5^ cells/well) were co-cultured with bacteria (MOI = 2) in triplicate wells of 24-well plates for 3 h, then bacteria were collected from supernatants resuspended by gentle pipetting, and CFU were determined by serial dilution and manual counting. Bacteria were incubated directly in MSC CM for 3 h in (**B**). In (**C**) synergistic killing of *S. aureus* by MSC CM and cefazolin. Cefazolin was added at a dose of 50 ng/ml and bacteria added at 1 × 10^6^ per well to 1 ml of MSC-CM or media alone in a 24 well plate and incubated for 3 hours at 37 °C, *denotes p < 0.05 as assessed by ANOVA and Tukey multiple means post-test. Synergy assessed via two way ANOVA for detection of significant interaction, as described previously^46^. Immunocytology was used to assess intracellular expression of the antimicrobial peptide Cramp by MSC in (**D**), as described in Methods. Cells were immunostained with an irrelevant antibody (isotype panel insert) or with an anti-Cramp antibody (**D**) and cells were evaluated by fluorescence microscopy. Synergistic killing between antimicrobial peptide LL-37 and beta lactam antibiotic demonstrated in (**E**). Human LL-37 at 30 ug/ml was incubated with 50 ng/ml Cefazolin for 3 hours with 1 × 10^6^ CFU/ml of S. *aureus*. In (**F**), MSC were co-cultured with bacteria and the effects of Cramp neutralization on bacterial killing were assessed Synergistic killing of bacteria was demonstrated between MSC and cefazolin (**C**) and between LL-37 and cefazolin (**E**). *Denotes p < 0.05 as assessed by ANOVA and Tukey multiple means post-test. Similar results were obtained in two additional experiments.

Previous studies have reported that MSC can kill multiple different species of bacteria directly, including *Klebsiella pneumoniae, Escherichia coli, Pseudomonas aeruginosa* and *Staphylococcus aureus* ^9, 12, 13, 19, 47, 57, 58^. To assess whether MSC or MSC-secreted factors could kill *S. aureus* directly in our system, MSC were incubated with *S. aureus* and the effects on bacterial viability determined 3 h later (Fig. 4). Incubation of bacteria with MSC resulted in significant direct killing of *S. aureus* (Fig. 4A). Moreover, conditioned medium (CM) from MSC cultures also elicited significant bacterial killing (Fig. 4B), suggesting that MSC secreted factors were primary mediators of the rapid, MSC-mediated antibacterial activity. However, it is important to note that MSC activation with poly I:C did not increase the antibacterial activity (data not shown). Thus, MSC constitutively produced factors with antibacterial activity, and TLR activation was not required to induce production of these factors.

Results from the mouse chronic implant infection model revealed a strong interaction between activated MSC and antibiotics in terms of eradicating bacteria in chronically infected tissues (see Fig. 1). Therefore, we next investigated whether MSC secreted factors could potentiate the bactericidal activity of antibiotics. To assess this interaction, MSC cultures were inoculated with *S. aureus*, alone or together with sub-therapeutic concentrations of the beta-lactam antibiotic cefazolin, and the effects on bacterial viability were analyzed (Fig. 4C). These studies revealed that when bacteria were exposed to MSC or to MSC CM, they became more susceptible to antibiotic killing. This significant enhancement of antibiotic activity by MSC was also observed with other classes of antibiotics, including penicillins, aminoglycosides, carbapenems, and fluoroquinolones (data not shown). This phenomenon suggested a general, non-specific mechanism of enhancement of antibiotic activity by MSC produced factors.

Antimicrobial peptides, including cathelicidins, are secreted by a number of cell types including MSC and are capable of direct bacterial killing, typically by induction of membrane pores^59^. One cathelicidin (CAP-18 or LL-37 in humans) or cathelicidin-related antimicrobial peptide (also referred to as CRAMP in mice), has previously been identified as a mediator of MSC-elicited bacterial killing in human MSC^9^. Therefore, we investigated the role of CRAMP in MSC-induced killing of *S. aureus* in our system. First, it was determined using immunocytochemistry with an anti-CRAMP antibody that murine adipose-derived MSC expressed intracellular CRAMP **(**Fig. 4D). In addition, CRAMP neutralization studies revealed that a significant portion of MSC *S. aureus* killing activity was CRAMP-dependent **(**Fig. 4F). Next, it was determined that incubation of *S. aureus* with sub-lethal concentrations of LL-37 sensitized the bacteria to killing by cefazolin (Fig. 4E). CM from MSC also induced synergistic killing of *S. aureus* when combined with cefazolin and other classes of antibiotics (data not shown). These results suggest that the demonstrated synergistic control of bacterial infection observed following *in vivo* administration of activated MSC is mediated in part by the local interaction of MSC-secreted antimicrobial peptides such as CRAMP with antibiotics at the wound site.

### Stimulation of neutrophil phagocytosis by activated MSC

**Figure 5:**
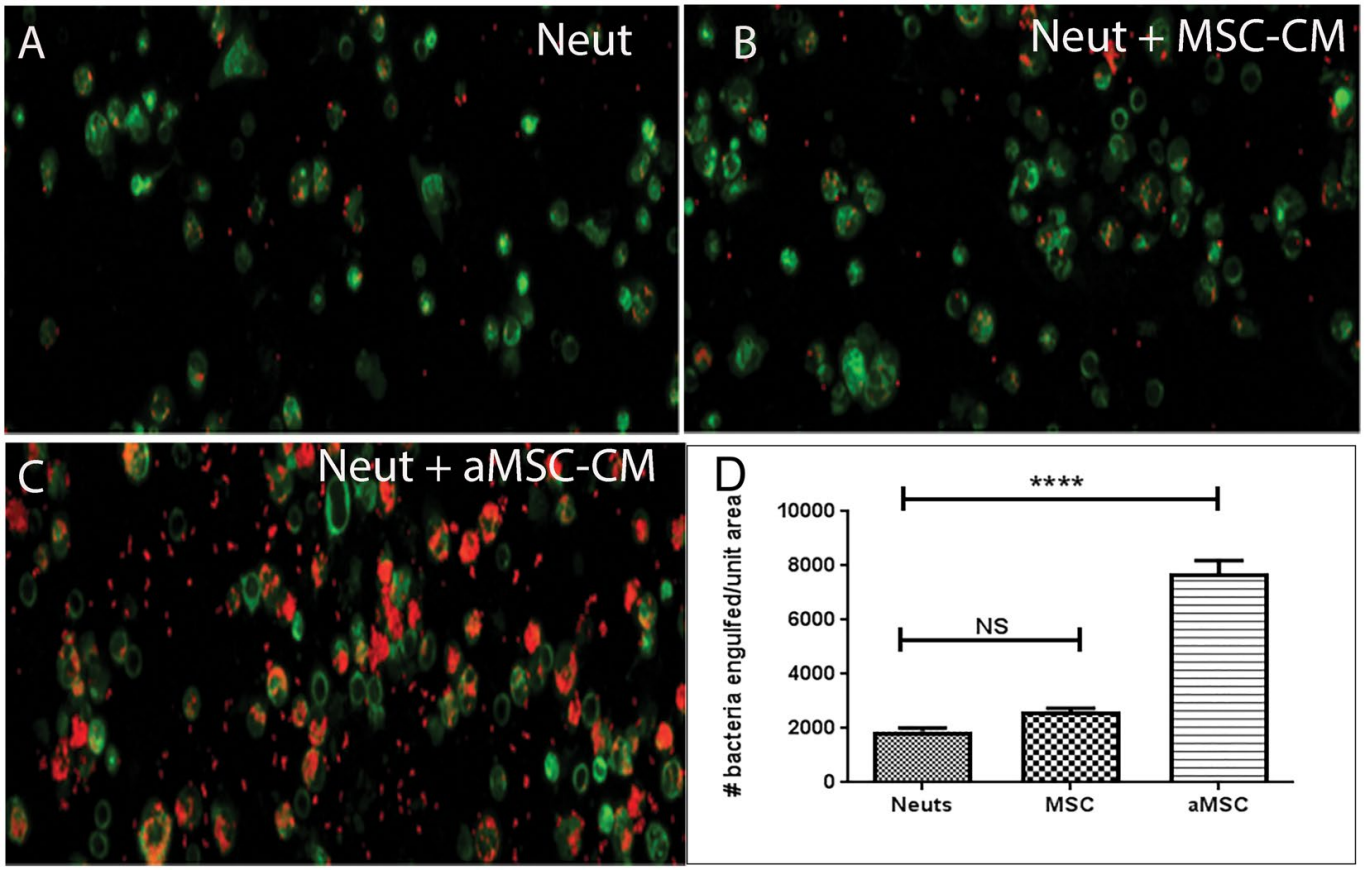
Incubation of neutrophils with MSC-CM increases phagocytosis of S. aureus. The effect of MSC secreted factors on neutrophil phagocytosis was assessed by first incubating mouse neutrophils (derived from peritoneal lavage as noted in Methods), then assessing neutrophil phagocytosis of *S. aureus*. Following incubation neutrophils were infected with *S. aureus* at an MOI of 1 for 30 minutes and extracellular bacteria was killed by the addition of gentamycin. Neutrophils were stained with CD11b (green) and S*. aureus* stained with anti-staph antibody (red). Neutrophils incubated with medium alone (**A**), resting MSC-CM (**B**) or activated MSC-CM (**C**). Image analysis was done using Image J software (NIH). Statistical analysis was performed using way ANOVA and Newman-Keul post test *indicates p < 0.05. Similar results were obtained in two additional experiments.

The preceding studies established that MSC were capable of killing *S. aureus* directly by producing antimicrobial peptides, but these studies did not however rule out a contribution of additional indirect effects of MSC leading to overall control of bacterial infection *in vivo*. In particular, activation of host innate immune defenses by systemically administered MSC would be a likely candidate for additional mechanisms of MSC antimicrobial action *in vivo*. To investigate the interaction of MSC with host innate immune responses, we first assessed the effects of MSC on neutrophil activity. Neutrophils recovered from the peritoneal cavity of mice were incubated with CM from poly I:C-activated and non-activated MSC to assess effects on bacterial phagocytosis. It was observed that CM from MSC significantly increased neutrophil phagocytosis of *S. aureus* and that this phagocytosis-enhancing effect was increased when neutrophils were incubated with CM prepared from poly I:C-activated MSC (Fig. 5A–D). These data indicate that MSC can release factors that increase the antibacterial activity of neutrophils. The effects of MSC on neutrophil antibacterial activity may be enhanced by systemic administration, as compared to local or topical administration, due to increased potential for interaction with immune cells present in circulation or in tissues.

### Monocyte recruitment and macrophage differentiation in wounds in response to MSC administration

**Figure 6:**
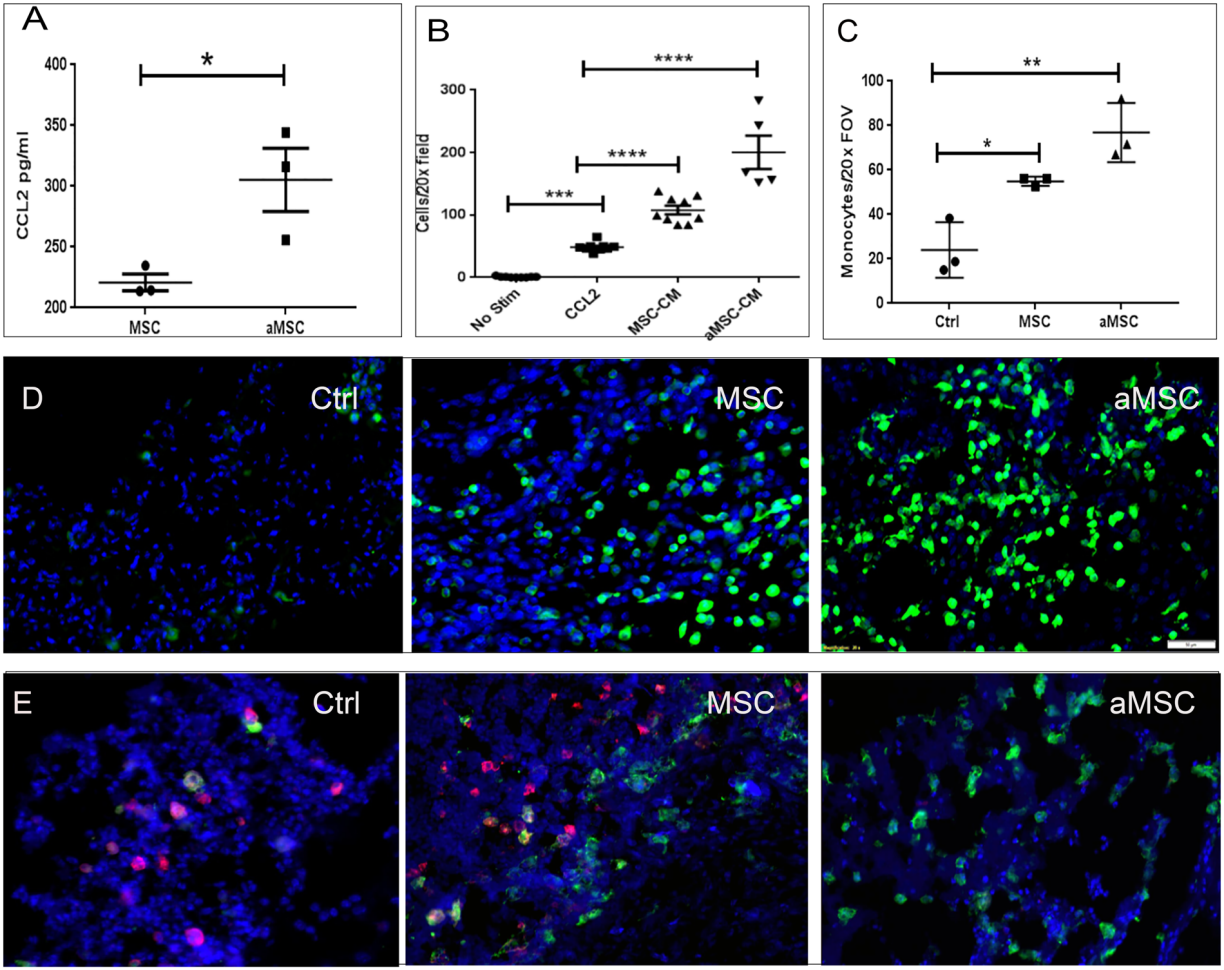
Mesenchymal stem cells increase monocyte migration and change the phenotype of macrophages in tissues. Production of CCL2 by MSC was measured in CM from resting MSC and pIC activated MSC (**A**). The effects of MSC CM on monocyte migration was assessed using Boyden chambers and monocytes isolated from the peritoneal cavity of thioglycollate-treated mice, as described in Methods. Cell migration data was quantitated using Cell Sens software, Olympus). MSC-CM significantly stimulated monocyte migration compared to medium alone, and CM from activated MSC triggered significantly more migration than CM from non-activated MSC (**B**). In mice treated with MSC *in vivo*, monocyte recruitment to infected wounds was assessed using CCR2-GFP reporter mice. Monocytes were present in relatively small numbers in tissues of untreated mice (**D**), whereas the numbers of infiltrating monocytes were significantly higher in mice treated with MSC and further increased in mice treated with activated MSC. These results were verified using tissue samples previously collected from non-GFP reporter mice at the same timepoint. In addition to altering monocyte migration, phenotype of tissue macrophages was also altered in the three groups of mice (control, MSC, aMSC). In tissues of untreated mice (Ctrl) the macrophages were mostly of the M1 phenotype (**E**) and fluoresced in the red channel.  Mice treated with resting MSC (MSC) had a combination of M1 and M2 (green) macrophages and activated MSC (aMSC) contained mainly M2 macrophages. Monocytes were stained with F4/80, anti-iNOS antibody, and anti arginase antibody. The cells were quantified using Cell Sens software, Olympus) Statistical analysis performed using ANOVA with Tukey multiple means comparison, *denotes p < 0.05.

In addition to neutrophils, monocytes and macrophages play a key role in innate immune control of wound infections. Therefore, the impact of MSC administration on monocyte and macrophage responses in wound tissues was evaluated (Fig. 6). *In vitro*, MSC were found to produce significant amounts of the chemokine CCL2, which is the major chemokine responsible for recruitment of inflammatory monocytes^60^. Significantly more CCL2 was produced by activated MSC (Fig. 6A). Migration of monocytes isolated from the peritoneal cavity of mice to CM from poly I:C activated and resting MSC (aMSC-CM and MSC-CM, respectively) was assessed using Boyden chamber migration assays. Conditioned medium from activated MSC induced significantly greater monocyte migration than CM from non-activated MSC (Fig. 6B).

To determine whether MSC administration triggered monocyte recruitment to infected tissues *in vivo*, CCR2-GFP reporter mice were used to track the migration of inflammatory monocytes expressing CCR2, a marker of inflammatory monocytes^42, 60^. Mice with *S. aureus* infected implant infections were injected i.v. twice with MSC (3 days apart), and 24 hr later the recruitment of gfp + inflammatory monocytes to infected tissues was assessed using immunohistochemistry and confocal microscopy (Fig. 6D). A marked infiltrate of CCR2-GFP^+^ monocytes was observed in infected tissues of mice injected with both non-activated and activated MSC. Consistent with the *in vitro* migration data, the numbers of CCR2^+^ monocytes was significantly greater in infected tissues of mice treated with activated MSC than in mice treated with non-activated MSC or in control animals (Fig. 6C).

Inflammatory monocytes rapidly differentiate into macrophages once they enter inflamed tissues^61^. In tissues, these macrophages can be classified as either M1 or M2 macrophages based on their cytokine and biochemical profiles, and it is known that macrophages with an M2 phenotype play an important role in wound healing^62^. For example, M2 macrophages, characterized in part by expression of the enzyme arginase, promote wound healing by secretion of growth factors including VEGF and EGF that stimulate wound angiogenesis and epithelialization, respectively^63^. In contrast, M1 macrophages produce factors such as nitric oxide synthase and function primarily to generate antimicrobial activity^64, 65^. Mesenchymal stem cells have been shown previously *in vitro* to secrete factors that regulate macrophage phenotype and stimulate macrophage M1 to M2 differentiation^33^. However, the effects of MSC administration on macrophage phenotypes in chronically-infected tissues have not been previously investigated. Therefore, macrophage phenotypes in infected tissues was assessed, using iNOS and arginase expression to identify M1 and M2 macrophages, respectively. We found that the majority of macrophages in untreated infected tissues expressed an M1 phenotype (iNOS^+^), while in infected tissues from mice treated with activated MSC, most macrophages expressed an M2 phenotype (arginase^+^). Macrophages from infected tissues of animals treated with non-activated MSC expressed a mixed phenotype of iNOS^+^ and arginase^+^ macrophages (Fig. 6E). Quantitatively, infected tissues from mice treated with non-activated MSC contained a roughly equal proportion of M1 and M2 macrophages, while the ratio of M2 to M1 macrophages was significantly increased in infected tissues of mice treated with activated MSC (Fig. 6E). Infected tissues with greater numbers of M2 macrophages would be expected to heal more effectively, consistent with the physical and histological appearance of infected tissues from mice treated with activated MSC (see Fig. 2). These findings indicate that MSC can interact extensively with the host innate immune system during tissue infection and healing, and this interaction likely contributes substantially to the overall antimicrobial effects observed in MSC-treated animals.

### Clinical evaluation of MSC therapy dogs with chronic infections with MDR bacterial

**Figure 7:**
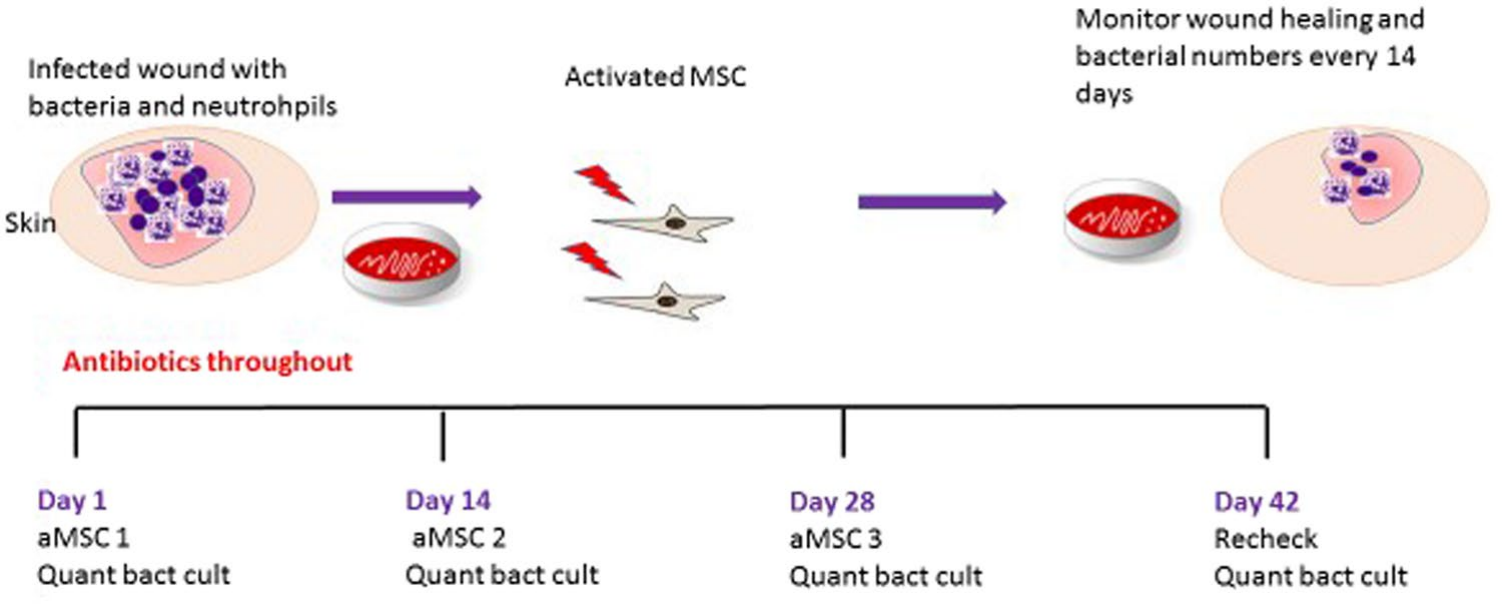
Illustration of Study Design. Dogs with naturally occuring infections were selected for treatment with activated canine allogeneic MSC according to inclusion and exclusion criteria explained in methods. The wounds were quantitatively cultured prior to the start of treatment and every 2 weeks prior to MSC injection. Dogs were evaluated 2 weeks after the last treatment and a culture performed at that time. Dogs were kept on the antibiotic they were taking prior to enrollment in the trial for the length of the trial.

A pilot study of MSC antimicrobial therapy was conducted in pet dogs with spontaneously-occurring, multi-drug resistant (MDR) infections to determine whether the observations from the mouse implant infection model could be translated to a realistic large animal model (Fig. 7). The pilot study enrolled 7 pet dogs that were initially evaluated at the Colorado State University Veterinary Teaching Hospital for management of chronic, non-responsive infections containing MDR strains of bacteria. Study animals included the following: 3 dogs with post-operative stifle infections, one dog with an infected traumatic foot wound, one dog with infected pacemaker leads, one dog with an infected bone plate and osteomyelitis, and one dog with deep pyoderma (Table 1). All of the animals had been extensively pre-treated with antibiotics (for at least 2 weeks) prior to study entry, without evidence of treatment responses.

**Table 1:** Patient data from 7 pet dogs with spontaneous, chronic infections with MDR bacteria treated with activated MSC.

Dog	Infection site	Infection duration	Organism(s)	Bacteriologic response (8 wks)	Clinical Response (8 wks
1	Post-operative stifle infection	12 months	MRSP	Eliminated	Resolved
2	Post-operative stifle infection	6 mos	MRSP	Eliminated	Resolved
3	Draining tract stifle	4 mos	MRSP	Eliminated	Resolved
4	Soft tissue injury- paw	4 weeks	PA, EC	Eliminated	Resolved
5	Infected bone plate	3 mos	MRSP, EC, Crny, Kleb	Eliminated (except MRSP)	Improved
6	Cervical abscess from pacemaker lead	24 mos	MRSP- 2 strains	Unchanged	Improved
7	Deep pyoderma - paws	9 mos	MRSP	Eliminated	Resolved

PA = *Pseudomonas aeruginosa*, EC = *Eschericia coli*, MRSP = methcillin resistant *Staphylococcus pseudointermedius*, Crny = *Corynebacterium sp*, Kleb = *Klebsiella sp*.

The 6-week MSC antimicrobial study protocol is described in Fig. 7. Briefly, study animals were required to remain on the original ineffective antibiotic during the entire 6-week study. Quantitative bacterial cultures were obtained from multiple aspirates of infection sites (except joints, where a single sample was obtained) prior to initiating treatment with MSC, and at 2-week intervals thereafter. Each animal received a series of 3 infusions of allogeneic, poly I:C-activated canine MSC derived from adipose tissue of healthy young, purpose-bred donor dogs. Each treatment consisted of a slow i.v. infusion of cells over 15 minutes via a peripheral vein catheter, at a dose of 2 × 10^6^ cells per kg body weight. Infection site healing was assessed by serial photographs of the lesions, or in the case of joint infections, by serial cytologic examination of synovial fluid samples.

Infusions of activated allogeneic MSC were well-tolerated clinically by study animals, with no notable adverse effects noted during or after infusion. In one dog (dog #6), the owner felt that the dog was more lethargic for 24 hours following infusion of MSC. As the dog had a history of cardiac dysrhythmias the dog was hospitalized for 24 hours following the next i.v. infusion with EKG monitoring, and arrhythmias or behavioral abnormalities were not noted while the dog was under observation. It was concluded that the stress associated with evaluation and treatment may have been responsible for the lethargy following the first MSC infusion. In 2 of the 3 dogs with stifle infections (Dogs 1 and 2; Table 1), signs of infection (lameness, pain on joint palpation) were completely resolved by the completion of the 6-week study period. Clinical improvement was first apparent within 2 weeks of MSC infusion in the treated dogs, and further improvement was noted after the second and third infusions in dogs 1 and 2. Dog 3 had persistence of lameness although pain on palpation of the stifle resolved. In dog 3, the final cause of lameness was determined to be persistent flexion of the joint and tendon contracture. After institution of physical therapy in dog 3, clinical improvement was noted. In dogs 1 and 2, there was no evidence of infection recurrence or need for additional treatment for more than 12 months of follow-up. Dog 3, which originally had a draining tract presumably from the stifle joint, had recurrence of infection 4 months after completing the MSC treatment. In this instance, a different organism was isolated (*Acinetobacter)*. This dog subsequently underwent surgery and had the stifle implant removed, and this dog has done well in the last 4 months of follow-up.

**Figure 8:**
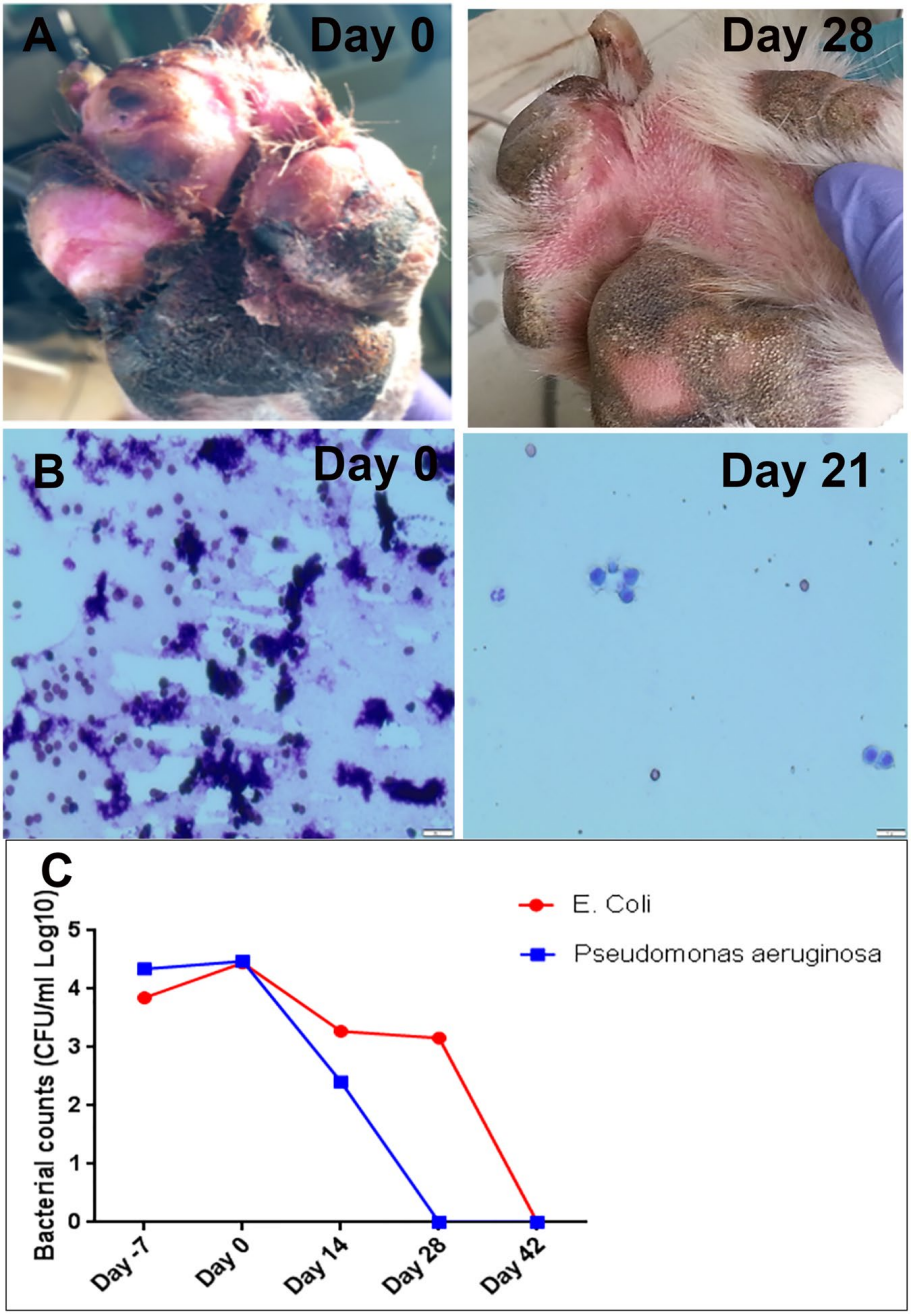
Effects of antimicrobial treatment with activated allogeneic canine MSC in pet dogs with spontaneous wound infections with MDR bacteria. Pet dogs with chronic wound infections unresponsive to prolonged antibiotic therapy were enrolled in a clinical trial designed to evaluate the effects of systemic administration of activated canine MSC, as described in Fig. 7. In (**A**) photographs of the paw wound in a dog before and 4 weeks after MSC treatment. In (**B**) serial cytological evaluation of joint fluid samples obtained from a dog with septic arthritis prior to treatment and at week 4 of treatment with activated MSC. The dog developed post-operative septic arthritis with MDR S. pseudointermedius and had been unresponsive to several month long courses of antibiotic treatment over a period of 12 months. The animal became clinically normal at the completion of the 6 week study and the joint fluid remained cytologically normal.  In (**C**) serial quantitative cultures of a soft tissue infection from traumatic paw injury displayed in A shows resolution of two multidrug resistant infections throughout the treatment period.

Initial evaluation of synovial fluid samples from the affected stifle of dogs 1 and 2 revealed marked inflammatory cell infiltrates prior to treatment (Fig. 8A). Following treatment with activated MSC, serial evaluation of synovial fluid and revealed progressive reduction in overall cellularity and a shift from neutrophilic inflammation to moderate monocytic inflammation, and the synovial fluid was judged to be normal by 8 weeks after treatment (Fig. 8A). Dog 3 had cultures performed at the draining tract (by fine needle aspirates) on day 0 and on days14, 28, 42 days post treatment. All cultures became negative after the initial day 0 culture.

In Dog 4 (Table 1) with traumatic soft tissue infection of the paw, there was a progressive clearance of both strains of MDR bacteria (*E coli* and *Pseudomonas aeruginosa*) from the wound bed over time following MSC treatment (Fig. 8B). Both strains of bacteria were resistant to the treatment antibiotic (amoxicillin/clavulanic acid) throughout the 6-week study. The infected paw healed rapidly during the MSC treatment period (Fig. 8C).

Following MSC treatment in Dog 5 (Table 1; infected bone plate and osteomyelitis), 2 of the 3 MDR bacterial strains were eliminated, except for *S. pseudointermedius*. The infected bone plate site was clinically improved, though complete skin coverage of the exposed plate was not achieved. However, the dog was able to retain the bone plate until bone healing progressed enough to allow eventual removal of the implant at 22 months.

In the dog 6 (infected pacemaker leads for 2 years of continuous antibiotic treatment, Table 1), the infected pacemaker lead site became bacteriologically sterile following the initial 3 MSC treatments (data not shown), though the site did not fully heal. An additional 3 MSC treatments were administered, along with surgery to further debride the wound and remove a portion of the pacemaker lead, at which point the infection was resolved.

Study dog 7 (recurrent, deep pyoderma; Table 1) was treated with a series of 3 i.v. infusions of MSC, along with cephalexin. Prior to treatment, skin cultures revealed high growth of methicillin resistant *Staphylococcus pseudointermedius*. Following 3 i.v. administrations of MSC, skin cultures became negative within 6 weeks, and clinical signs of pyoderma resolved by the completion of the 8-week study. However, signs of pyoderma recurred 6 months later.

The spontaneous infections in dogs in this study, which are representative of similar chronic, MDR infections in humans, provided an important opportunity to assess the overall safety and potential effectiveness of activated systemic MSC therapy in a large animal model. This pilot study established the safety of activated MSC in dogs, and provided some evidence for efficacy, though it is acknowledged that a larger randomized clinical trial would be required to absolutely establish efficacy. A number of prior studies have established the safety of i.v. delivered allogeneic MSC for treatment of diverse conditions ranging from cardiac infarction to autoimmunity and chronic inflammatory disorders^66–68^. Moreover, the TLR3 agonist pIC has been administered safely to humans as a vaccine adjuvant and non-specific immunotherapeutic for^69, 70^. Therefore, we believe these results provide a strong rationale for the evaluation of TLR3 activated allogeneic MSC as a novel treatment for chronic, drug-resistant infections in humans.

## Discussion

Chronic infections, especially those associated with implanted foreign material such as catheters or metallic devices, remain a major problem clinically as these infections are very difficult to resolve with antibiotic therapy alone^71–74^. Our findings suggest that systemic administration of activated MSC, when combined with conventional antibiotic therapy, offers one solution to the problem of chronic, drug-resistant infections. Our studies revealed a strong positive interaction between activated MSC and conventional antibiotics, as well as between activated MSC and the host innate immune response to infection. The net result of the interactions between MSC and antibiotic therapy was significant clearance of bacteria even from heavily infected synthetic implant materials such as mesh coated with *S. aureus* biofilms. Moreover, MSC administration also produced other benefits, including apparent stimulation of wound healing and reduction of infection-associated inflammation and fibrosis.

Multiple, complementary mechanisms of action (both direct and indirect) likely account for the ability of activated MSC to help control wound infections. As has been reported by others, our studies showed that MSC secrete antimicrobial peptides^9–11^. We also identified indirect mechanisms of enhanced bacterial elimination by MSC involved interactions of activated MSC with the host innate immune response, including both neutrophils and monocytes. For example, activated MSC were noted to secrete factors which enhanced neutrophil bacterial phagocytosis, resulting in more effective bacterial killing. In addition, activated MSC stimulated monocyte recruitment to wounds and the differentiation of macrophages into an M2 phenotype, which has been associated previously with accelerated wound healing^63, 64^. Previous studies by our lab and others have also demonstrated that MSC can elicit recruitment of monocytes and macrophages into tissues^75, 76^ (Takeda K, Webb T and Dow S. manuscript submitted). Importantly, these neutrophil and monocyte effects were enhanced by pre-activation of the MSC in the current studies. The effects of activated MSC on monocyte recruitment and differentiation may play an important role in both antimicrobial activity and in stimulation of wound healing.

Despite the noted propensity for systemically administered MSC to lodge in the lung, in our study we observed that i.v. administration of MSC resulted in effective control of deep-seated bacterial infections in sites distant from the site of MSC administration^52^. Additionally we found lower bacterial counts in wounds from animals treated with intravenous administration of MSC when compared to subcutaneous administration (data not shown). The potential increased interaction of MSC with neutrophils and monocytes when administered i.v. as compared to local administration into wound sites may in part explain the greater efficacy of systemic MSC administration in these cases. For example, MSC were observed to accumulate in the spleen as well as in wound tissues following i.v. administration, and localization of MSC in secondary lymphoid organs such as the spleen may further enhance systemic activation of innate immune defenses. The presence of MSC in the spleen may also modulate the host adaptive immune response to bacterial infection (eg, stimulation of humoral immune responses to bacteria), though this issue was not specifically addressed in this study. Indeed, systemic administration of MSC may be critical to the activation of other immunomodulatory cells that play crucial roles in the wound healing process^75^.

While MSC injected i.v. lodged initially in the lungs as has been seen previously, *in vivo* imaging studies showed that substantial numbers of MSC migrated from the lungs to the wound site over the next 24–72 hours. The stimulus for MSC recruitment into wounds likely includes chemokines such as SDF-1, produced locally by inflamed wound tissues^53, 77^. In addition, activation of MSC with poly I:C facilitated MSC recruitment to sites of chronic infection, an effect that may be attributed in part to up-regulation of the receptor CXCR4, for which SDF-1 is the principal ligand. These findings demonstrate that i.v. injected MSC remain viable in the lungs and can migrate efficiently from this site to localize at infected wounds over a period of several days. The migratory potential of systemically administered MSC is particularly relevant for treatment of wounds in sites that cannot easily be reached for direct MSC injection (eg, bone infections, implant-associated infections).

In the era of increasing antibiotic resistance, there is a clear need for new, non-antibiotic approaches to controlling drug-resistant infections. Previous studies have determined that MSC secrete antimicrobial peptides such LL-37^10, 12, 58, 78, 79^. Other investigations have demonstrated that antimicrobial peptides, including cathelicidins, can augment the antimicrobial activity of certain antibiotics^80^. However, these earlier studies did not specifically examine the interaction between MSC-secreted factors and antibiotics with respect to augmented bacterial killing. Our assays revealed that the mouse cathelicidin CRAMP was one of the factors produced by MSC that significantly increased staphylococcus killing. Moreover, in other studies we have observed that MSC-secreted factors can reduce high level antibiotic resistance in bacterial strains with multi-drug resistance (Johnson V, manuscript in preparation). These findings suggest that activated MSC therapy would be particularly useful in the management of deep-seated infections with highly drug-resistant strains of bacteria, where treatment options other than antibiotic therapy may not exist.

The translational relevance of studies conducted in rodent models is not always assured. Accordingly, we completed a pilot study in dogs with spontaneous chronic wound infections where we observed wound infection clearance and healing in dogs treated with repeated i.v. infusions of activated allogeneic MSC, without evidence of toxicity or adverse effects. The apparent beneficial effect from MSC administration was observed in wounds in diverse locations (eg, joints, cutaneous wounds, infected surgical implants) and with diverse, highly drug-resistant strains of bacteria. These results suggest that systemic administration of activated, allogeneic MSC may be a viable option for the treatment of chronically infected wounds in other species such as humans. Allogeneic MSC have been safely administered to humans for a number of conditions previously, and their use as a treatment for chronic infections would not represent a unique risk^81, 82^. Patients with infected implants or other wounds associated with biofilm formation and highly drug resistant strains of bacteria (eg, infected ulcers in diabetic patients) that have failed conventional antibiotic therapy represent patient populations that may benefit from a short-course of systemic activated MSC therapy. As the need for non-antimicrobial solutions to management of chronic infections grows, use of activated MSC may represent a new solution for some patients with few other viable options.

### Summary

Activated MSC, when administered i.v. together with conventional antibiotics, can potently suppress and eradicate chronic *S. aureus* biofilm infection in difficult-to-treat locations. Injected activated MSC migrated to the site of wound infection, where they stimulated bacterial killing by secreting antimicrobial peptides and activating host innate immune defenses. These studies suggest that therapy with activated MSC represents a novel option for treatment of patients with highly drug-resistant infections and no ready surgical or medical options for infection control.
